# On the Potential of the RST-FLARE Algorithm for Gas Flaring Characterization from Space

**DOI:** 10.3390/s18082466

**Published:** 2018-07-30

**Authors:** Mariapia Faruolo, Teodosio Lacava, Nicola Pergola, Valerio Tramutoli

**Affiliations:** 1 Institute of Methodologies for Environmental Analysis, National Research Council, 85050 Tito Scalo, Italy; teodosio.lacava@imaa.cnr.it (T.L.); nicola.pergola@imaa.cnr.it (N.P.); 2 School of Engineering, University of Basilicata, 85100 Potenza, Italy; valerio.tramutoli@unibas.it

**Keywords:** gas flaring, remote sensing, MODIS, RST-FLARE, BTE, flaring sites detection, gas flared volumes computation

## Abstract

An effective characterization of gas flaring is hampered by the lack of systematic, complete and reliable data on its magnitude and spatial distribution. In the last years, a few satellite methods have been developed to provide independent information on gas flaring activity at global, national and local scale. Among these, a MODIS-based method, aimed at the computation of gas flared volumes by an Italian plant, was proposed. In this work, a more general version of this approach, named RST-FLARE, has been developed to provide reliable information on flaring sites localization and gas emitted volumes over a long time period for the Niger Delta region, one of the top five gas flaring areas in the world. Achieved results showed a good level of accuracy, in terms of flaring sites localization (95% of spatial match) and volume estimates (mean bias between in 16% and 20%, at annual scale and 2–9% in the long period) when compared to independent data, provided both by other satellite techniques and national/international organizations. Outcomes of this work seem to indicate that RST-FLARE can be used to provide, at different geographic scales, quite accurate data on gas flaring, suitable for monitoring purposes for governments and local authorities.

## 1. Introduction

Gas flaring is one of the processes, together with venting and reinjection, widely used to dispose of natural gas produced at oil and gas facilities, especially in situations where there is insufficient infrastructure to use the gas locally or move it to market [1]. It represents a foremost anthropogenic source for greenhouse and/or precursor gases, particulate matter and black carbon [2,3] that pollute the air, soil and water, with huge impacts on humans, the environment and the economy [3,4,5,6,7]. The World Bank estimates that over 150 billion cubic meters (BCM) of natural gas is flared annually, an amount worth approximately 30.6 billion dollars, equivalent to 5% of the world’s natural-gas consumption [8] and causing 400 million tons of carbon dioxide (CO_2_) discharged into the atmosphere [9].

Nevertheless, oil companies continue to annually flare billions of cubic meters of natural gas. New data recently released by Global Gas Flaring Reduction Partnership (GGFR) for 2016 revealed an increase of 2 BCM, from the previous year (i.e., 147 BCM), in the amount of gas flared at oil production sites worldwide, reversing a general trend of gas flaring reduction, observed in the previous two years [10]. Such an increase is mainly attributed to an overall growth in oil production, particularly in Russia, Iran and Iraq [10]. In the rest of the world, especially in the United States and Nigeria, gas flaring has significantly declined in the past two years while oil production levels have remained unchanged [10,11].

The main challenges involved in addressing environmental aspects of flaring consist in recognizing the most affected areas worldwide and determining how much emission is actually released. Until recently, several factors have hampered an empirical assessing of the environmental impacts of flaring: (i) a limited access to official information on flaring locations; (ii) the heterogeneity in spatial and temporal sampling strategies; (iii) the methods used to collect data and (iv) the lack of auditability [7,12]. Furthermore, even when data on gas flared volumes are available, they typically are: (i) self-reported by the flare operators and (ii) roughly estimated on the basis of the difference between the natural gas volume produced and the quantity used or sold.

The difficulty in assessing the reliability and accuracy of the reported data [12] points out the need of developing robust, consistent and objective methodologies of data analysis able to provide independent information about active flaring sites localization, the amount of gas flared volumes at both site and country scale and flare volumes changes over space and time [12].

A valid way to bridge this gap and to have independent information on gas flaring activity at global, national and regional scale might come from the use of Earth Observation (EO) satellites, owing to their continuous and repetitive observations, multispectral capabilities and synoptic coverage [12,13]. In particular, the implementation of methodologies based on the use of long-term time series of satellite data has demonstrated its potential and usefulness for this aim. In detail, satellite-based methods allow to map and monitor the distribution of flaring sites over a large area, both in night-time [12,14,15,16] and daytime conditions [17,18], and to characterize such a phenomenon in terms of emitted gas volumes [12,16,19,20], consumption of CH_4_ and release of CO_2_ [21].

Most of the satellite techniques proposed and currently applied for detecting gas flaring platforms as well as to assess their spatiotemporal variations [7,12,16,17,21,22,23,24,25] are based on measurements acquired by different sensors (e.g., Operational Linescan System—OLS, Moderate Resolution Imaging Spectroradiometer—MODIS, Visible Infrared Imaging Radiometer Suite—VIIRS, Along-Track Scanning Radiometer—ATSR, Land Remote-Sensing Satellite—Landsat) in different portions of the electromagnetic spectrum (visible—VIS, near-infrared—NIR, shortwave infrared—SWIR, middle Infrared—MIR, thermal infrared—TIR). Considering that gas flares typically operate at temperatures >1450 K with a mean of around 1750 K [7,20,21], their peak spectral radiant output occurs in the SWIR spectral region, centered around 1.6 µm [7,25,26]. The latter is then the most suitable spectral region to be used for investigating these phenomena. On the other hand, when sensors not able to acquire information in this region are used, like MODIS in nighttime conditions, the MIR bands (centered around 3.5 µm) are the most used [7]. This portion of the electromagnetic spectrum falls on the trailing edge of gas flare radiant emissions, therefore observes a mixture of flare plus background (always contributing to the signal) radiant emissions [7]. Flare radiant emissions at these wavelengths are indeed about one third of the emissions at 1.6 μm, as suggested by [7] and [26], hence, for high temperature gas flares, a significant proportion of flaring activity is not observable above the MIR background [7]. Once selected the most suitable channel(s), gas flares can be separated from other IR emitters based on temperature and temporal persistence (i.e., a presence at the same location on multiple observations over a set period) [7,23,24,25].

With reference to the estimation of gas flared volumes, a regression model is usually applied [7,16], requiring, for its calibration, a diagnostic parameter aimed at quantifying the emission power of the source (e.g., the sum-of-lights index for OLS data—[16], the Fire Radiative Power for MODIS—[20] and VIIRS [26] data or the monthly sum of cloud and sampling normalized radiant heat estimates for VIIRS data—[7]) and official data on volumes of gas flared. It is worth noting that actual improvements in this field would be achieved by using multiple satellite data sources and when reliable sources of in situ measurements of gas flaring volumes for calibration would be available.

Among the few existing algorithms, ad hoc developed for gas flaring analysis, a new methodology has been recently proposed by [20] for computing the volumes of gas annually flared by a specific plant (i.e., Centro Olio Val d’Agri—COVA), located in southern Italy, through the analysis of time series of night-time MODIS data acquired in the infrared region. Such a methodology provided accurate estimates (R^2^ ~ 83%) of the volumes of gas emitted by COVA on an annual basis, with an error of about 2%. Starting from these encouraging outcomes, in this paper, the field of applicability of such a method has been extended further, adding a preliminary step aimed at the detection of areas affected by gas flaring activity. By this way, a more general approach is here proposed, named RST-FLARE. Such a method is described in this paper and its performances, achieved while analysing the Niger Delta region, are shown and discussed. Respect to the previous application (i.e., COVA) the use case here presented is significantly different as: (i) the amount of gas flared is dramatically higher; (ii) the number of flaring sites is enormously larger; (iii) the flaring site locations are not “a priori” known.

The accuracy of the achieved results will be evaluated for comparison with other satellite products; with respect to the gas flared volumes, the information arising from official datasets independently provided by international/national organizations will be also considered. Besides, owing to the availability of the long-term satellite data used, the RST-FLARE capability to reconstruct and monitor, in the spatiotemporal domain, the evolution of gas flaring activity in the investigated area, both in terms of areas affected by flaring sites and emitted volumes, will be assessed by considering independent sources of information.

The paper is organized as follows. Section 2 describes both the satellite (Section 2.1) and ancillary (Section 2.2) datasets used in the analyses, and the developed methodology (Section 2.3). In Section 3 the results achieved applying RST-FLARE for flaring sites detection (Section 3.1), sources emissive power estimation (Section 3.2) and gas flared volumes computation (Section 3.3) are shown and discussed. In Section 4 a validation is carried out by comparing RST-FLARE outcomes to data provided by other sources to assess their accuracy. Finally, Section 5 and Section 6 present discussion and conclusions, respectively.

## 2. Data and Methods

### 2.1. Satellite Data

**Figure 1:**
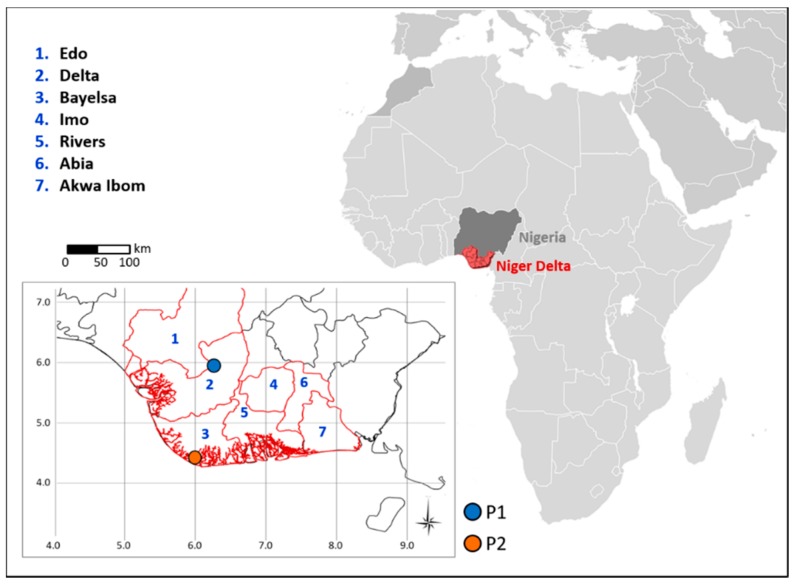
The Niger Delta region (red borders). Within the map, the seven states analyzed in this work are numbered and named. P1 and P2 are two locations used in the analyses (see text).

Daily Level 1B MODIS data acquired both from Terra and Aqua satellites from March 2000 to December 2016 over Nigeria in night-time conditions were used, for a total of 16.473 images (8.932 from Terra and 7.541 from Aqua), collected at monthly scale. These data were downloaded from the NOAA web site archive (https://www.class.ngdc.noaa.gov). The middle and thermal infrared bands of the sensor, channels 20 (3.660–3.840 μm), 31 (10.780–11.280 μm) and 32 (11.770–12.270 μm), respectively, were processed in terms of Brightness Temperature (BT), considering, as Region of Interest (ROI), an area of 600 pixel × 400 lines including the seven states of the Niger Delta region (Figure 1), re-projected in Lat/Long (WGS-84).

The satellite dataset, used in all analyses, consists of clear (i.e., not cloudy) pixels, identified by applying the OCA approach [27,28] based on MODIS channel 32 (11.770–12.270 μm) and acquired with a satellite zenith angle (SatZA) lower than 40° and solar zenith angle (SolZA) less than 101° to assure nighttime conditions. Due to these filters, considering the double impact of clouds and sensor viewing geometry, for each year (from 2000 to 2016) only a low percentage (ranging from 12% to 30%, with a mean value of 22%) of images available for the ROI resulted useful. However, due to the huge amount of original MODIS imagery, all the considered yearly datasets still remain sufficiently and homogeneously populated to guarantee a statistical significance of the achieved results.

### 2.2. Ancillary Data

Ancillary data were required to both develop the regression models and validate the results.

**Figure 2:**
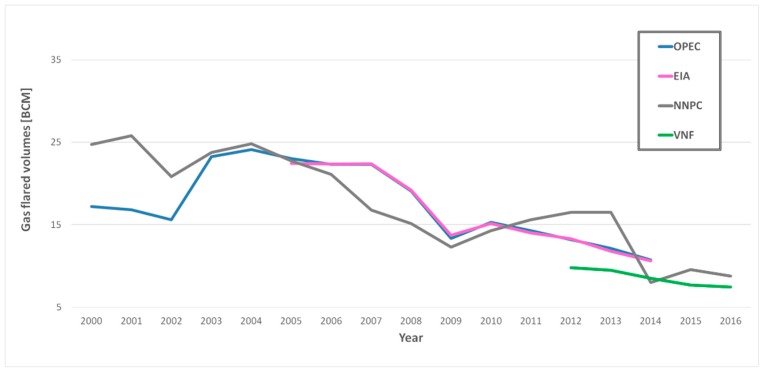
Annual values of gas flared volumes (in BCM) for Niger Delta region provided by NNPC (grey line), OPEC (blue line), EIA (pink line) and VNF (green line).

The volumes of gas flared in Nigeria (Figure 2) were derived from three different official datasets, made available online by: (i) Nigerian National Petroleum Corporation (NNPC), providing information from 2000 to 2016 [29]; (ii) Organization of the Petroleum Exporting Countries (OPEC), from 2000 to 2014 [30]; and (iii) Energy Information Administration (EIA), whose data cover the period 2005–2014 [31]. NNPC, OPEC and EIA are international organizations providing monthly or annual statistical bulletins, containing useful information on the oil and gas activities of their members as well as most of the worldwide oil-producing countries. Such data, of which they do not guarantee a 100% accuracy, reflect general analysis and trend of activities that characterize the industry within each country for a specific year. It is worth noting that the NNPC and EIA data were originally provided in mscf (million cubic feet) and bscf (billion cubic feet), respectively. In this study they were converted in Billion Cubic Meters—BCM (1 cubic feet = 0.0283168 cubic meter) to make possible the comparison among all official data. In detail, as shown later, OPEC and EIA were used to develop the regression models, while the NNPC records, offering the longest temporal range, were employed to validate the estimates of gas flared volumes.

Moreover, results obtained by another independent, MODIS-based, algorithm developed by [12] (i.e., MODET–MODIS Flare Detection Technique and MOVET–MODIS Flare Volume Estimation Technique) were also used for assessing the reliability of the RST-FLARE achievements, in terms of flaring sites localization and gas flared volumes estimation.

Besides, satellite products provided by NOAA’s Earth Observation Group (EOG), applying the VIIRS Nightfire algorithm–VNF [22], have been also exploited. Such data are made available as of November 2017 by SkyTruth, which has built an interactive map showing individual flaring locations as well as annual estimates of gas flaring volume over virtually any area of interest, for years 2012 through 2016 (https://viirs.skytruth.org/apps/heatmap/flarevolume.html). In this paper, the data referred to Niger Delta were extracted and used (green line in Figure 2).

### 2.3. RST-FLARE

RST-FLARE is a specific configuration of the Robust Satellite Techniques approach (RST, [32,33,34]), a general automatic change detection scheme widely and successfully applied to monitor the major environmental, natural and industrial hazards (e.g., [35,36,37,38,39]). In its original version, RST-FLARE was developed for computing the amount of gas annually flared by the COVA plant located in Basilicata region (southern Italy) [20]. A detailed description of this method can be found in [20]; here, the algorithm is briefly presented, providing more information about the new configuration proposed. To study and characterize the gas flaring phenomenon, RST-FLARE requires the implementation of three methodological steps, described in the following.

#### 2.3.1. Flaring Sites Detection

The difference between the BTs acquired in the MODIS MIR and TIR channels (i.e., bands 20 and 31) is investigated for describing the target behaviour. In detail, following RST prescriptions, the following ALICE_20_*_–_*_31_ index (Equation (1)) is implemented at the pixel level [20]:

(1) $${⊗}_{20-31}\left(x,y,t\right)=\frac{BT_{20-31}\left(x,y,t\right)-\mu_{20-31}\left(x,y\right)}{\sigma_{20-31}\left(x,y\right)},$$

where *μ*_20–31_(*x*,*y*) represents the value expected to measure for the signal under investigation (i.e., BT_20–31_) in unperturbed (or normal) conditions while *σ*_20–31_(*x*,*y*) is its natural variability, for the pixel located at (*x*,*y*) and acquired at time *t*. Both *μ*_20_*_–_*_31_(*x*,*y*) and *σ*_20_*_–_*_31_(*x*,*y*) (namely the reference fields) are computed, for each location (*x*,*y*), processing several years of historical satellite records acquired in homogeneous spatiotemporal conditions (e.g., same geographic area, same month and hour of acquisitions) of the image at hand. As assessed by an independent work focused on RST [40], such satellite historical series should consist of about 80 imagery to guarantee representative background signal. Such a number, considering a monthly temporal window and a sensor offering a daily temporal resolution, roughly corresponds to 3-year long time series, representing indeed the minimum time span for a reliable RST implementation. Longer time series will obviously allow to better facing cloud and satellite geometry view issues The robustness of the ALICE index is intrinsic, because any signal fluctuation due to natural and observational (known and unknown) sources produces an increase in the standard deviation reference field (i.e., *σ*_20_*_–_*_31_(*x*,*y*)), with a consequent reduction in the probability to detect an anomaly. Among these possible sources, the signal variability due to satellite viewing angles or atmospheric effects are also included, with the standard deviation accounting for these and protecting from possible false positives. According to RST configuration, gas flares are thermal features more difficult to discriminate, being generally persistent both in space and time. As investigated in [20], with reference to such targets, the ALICE index is expected to discriminate, among the measured radiant records, the most highly ones, associable to the major fluctuations in the combustion operations and/or to gas flaring in emergency conditions (extremely high and intense burst of flames are generally associated with these anomalous situations). Ref. [20] found that ALICE_20–31_ values ≥ 1.5 can be assumed as representative of thermal anomalies related to gas flaring, representing a significant signal deviation from what observed in normal operational conditions of the plant and likely related to unexpected events at the system, like accidents, malfunctions or extremely high oil production rates.

**Figure 3:**
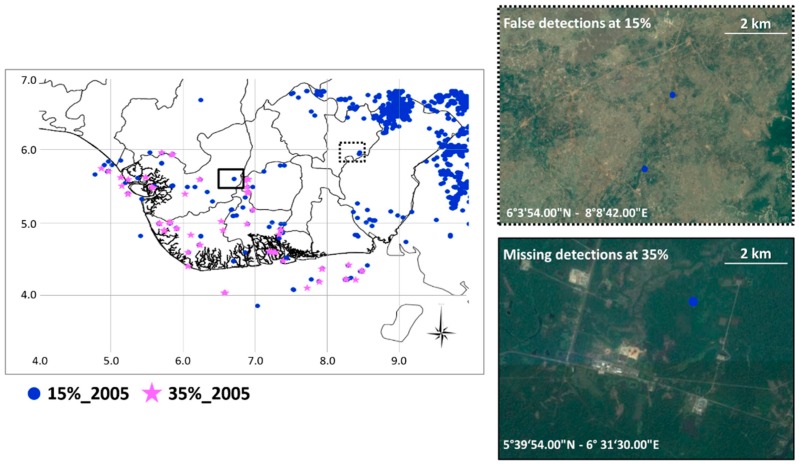
Flaring sites detected by RST-FLARE in 2005 for a temporal persistence percentage of 15% (blue dots) and 35% (pink stars). On the right a detail of false positives (**top**) and missed detections (**bottom**) are shown.

When the position of flaring sites is not known, a time persistency criterion has to be applied to the ALICE_20–31_ ≥ 1.5 to flag a pixel as flaring site, for discriminating such hot objects from other high thermally emitting sources, such as wildfires, which may also affect the Nigerian soils [41]. In particular, considering, for each year, the localization of the identified thermal anomalies, their percentage of recurrence within the reference annual dataset (i.e., only cloud-free pixels acquired with SatZA < 40° and SolZA < 101°) was computed. The algorithm classifies a pixel as potentially flaring-affected by applying a threshold to the annual recurrence percentage of the previously detected anomalies. Such a value is derived by a series of tests based on the comparison between potential persistent source maps, obtained at different recurrence percentage values and a reference map (i.e., Google Earth). The flaring site maps obtained using a percentage ranging from 15% to 35%, with a step of 5%, were analysed in comparison with the above mentioned reference. In Figure 3 an example of the two “extreme” maps for 2005 (randomly chosen within the investigated period) are shown.

**Figure 4:**
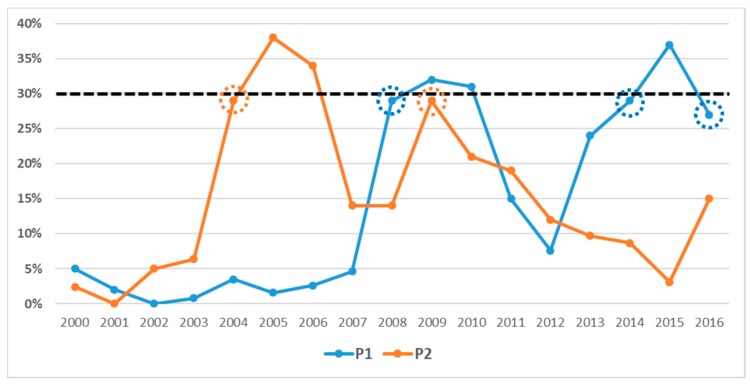
Temporal trends of the recurrence percentages computed for two pixels, P1 and P2, selected within the ROI (see Figure 1).

A high number of potential flaring sites was identified over the scene when the 15% recurrence percentage threshold was used (blue dots in Figure 3). Most of these sites is localized in the northeast corner of the scene (an example shown in Figure 3), mainly due to the (periodic) occurrence of wildfires in Nigerian areas [41], suggesting that a recurrence value of 15% is not adequate to discriminate the flaring sites, resulting in a consistent amount of false detections. On the contrary, the highest percentage level (i.e., 35%) seems to be too restrictive in the gas flares detection: by means of a visual inspection using Google Earth, some missed detections were observed (an example shown in Figure 3). The threshold, which has shown to assure the best trade-off between reliability (i.e., low false positive rate) and sensitivity (i.e., low false negative rate), resulted equal to 30%. Therefore, within each year, a pixel was classified as flaring site when thermal anomalies (ALICE_20_*_–_*_31_ ≥ 1.5) are detected over there at least in 30% of the images of the annual dataset (i.e., all clear pixels with SatZA < 40° and SolZA < 101°). At the end of this step, the algorithm provides, for each year, a list of candidate flaring locations. However, the availability of a multi-temporal dataset can allow further improving the accuracy of such a list, which might have missed intermittent active flares. At each site, in fact, the flaring process can vary in time due to the decommissioning of some flares as well as to their reactivation during a specific year or to the variation in the amount of oil produced. So, it can happen that an active flare is prevented to be detected only because the detection frequency is slightly lower than the defined threshold. Figure 4 shows an example of this occurrence for two pixels, named P1 (lat 5°52′32.25′′ N–lon 6°13′23.25′′ E) and P2 (lat 4°20′38.63′′ N–lon 5°55′26.62′′ E), arbitrarily chosen within the ROI (Figure 1).

They were not flagged as flaring sites in some years (i.e., 2008, 2014, 2016 for P1 and 2004 and 2009 for P2) only because their occurrence frequency was 29% (in 2008), 29% (in 2014) and 27% (in 2016), for P1 and 29% (for both 2004 and 2009), for P2.

Hence, such an under-sampling reduces the probability of detection and decreases the accuracy of flared gas estimates for flares with highly variable flaring activity. Owing to the long-term MODIS dataset analyzed, this issue can be faced mainly producing a multitemporal map of flaring locations. This map provides a global and general overview of areas affected by gas flaring activity in the whole 17-year period analysed. In fact, it considers as flaring sites all those pixels that, at least in one year (within the 17 investigated), have been detected as anomalous (i.e., ALICE_20–31_ ≥ 1.5) for 30% of the imagery available in the same year. Hence, by such a strategy, the RST-FLARE detection-devoted step allows for obtaining a twofold information about flaring locations: the annual-based map shows the flaring sites active “for sure” in that year while the multitemporal-based one, taking into account the activity variation of the site, highlights the areas affected by the gas flaring phenomenon in the whole 17-year period analyzed.

Finally, it is worth stressing that, in this paper, given the spatial resolution of MODIS data (i.e., 1 km^2^), with the term “flaring site” we do not refer to individual single gas flare but to a zone affected by the presence of oil processing facilities, that may possibly include a single or a group of flare systems.

#### 2.3.2. Source Emissive Power Computation

Unlike our previous study [20], in this case flaring sites are high temperature sources making the Fire Radiative Power (FRP) suffering for high uncertainties [26]. Thus, a new metric, named Brightness Temperature Excess (BTE), has been introduced to compute the emissive power of each flaring site. BTE is defined as the difference between the BT measured in the MODIS band 20 for the flaring site (BT20) on the image at hand and that of its surrounding historical background (BT20*_bg_*). The latter refers to the averaged value of the multi-temporal RST-based temporal mean (i.e., *μ*_20_(*x*,*y*)) computed for a 5 × 5 pixel window centered on the potential flaring site.

The BTE is computed for each flaring site only when a thermal anomaly has been identified by Equation (1). In order to better assess the impact of gas flaring in the area at national level, the BTE is assessed, at yearly temporal scale, for all pixels identified as flaring sites by the multitemporal scheme (regardless of their recurrence percentage) (Equation (2)). Moreover, to account for sampling and cloud coverage across years, the BTE value is corrected by a scaling factor *α*, depending on the number of useful images. The sum of such values for each investigated year provides a measure of the emissive power of these sources, which is expected to be related to the amount of gas burned off by the plant in a specific year.

In detail, BTE is computed using the following formula:

(2) $$BTE_{y}=\sum_{j=1}^{M}\left(BT20_{j}-<BT20_{j,bg}>\right)\cdot \alpha_{j,y}\left[K\right],$$

where:

- *y* refers to each of the investigated years in the 2000–2016 period;
- *j* refers to each of the M identified flaring site (only in presence of detected thermal anomaly);
- *BT*20*_j_* is the brightness temperature (K) measured in the MODIS band 20 for the target *j* (i.e., the detected flaring site);
- <*BT*20*_j_*_,*bg*_>, referred to the background, is equal to the spatial mean of the temporal mean *μ*_20_(*x*,*y*) in a 5 × 5 box around the site;
- *α* is a coefficient which weights the variability, between years, in sampling and cloud cover. For each of the detected flaring sites within the single year, *α_j_*_,*y*_ is computed as the ratio between the useful images (i.e., clear and with SatZA < 40° and SolZA < 101°) and the total ones. To take into account of *α_j_*_,*y*_ variability in the considered period, it has been normalized with respect to its maximum value computed within the 17 years (*α_y,max_* = 0.30).

#### 2.3.3. Gas Flared Volumes Estimation

A linear regression model coupling BTEs and official data on gas flared volumes is developed for assessing the amount of gas burned off by the hot sources within the studied area, on an annual basis. Gas flared volumes, with units of BCM, are calculated using Equation (3):

Gas flared_y_ = γ*BTE_y_ + β, (3)

where gas flared (assessed for the year *y*) is the response variable in the model (Y) and BTE the predictor variable (X); γ and β are, respectively, the regression coefficient and the Y-intercept. The latter are site-specific coefficients derived and valid for the Niger Delta region.

## 3. Results

Results achieved applying the RST-FLARE approach on the Niger Delta region will be presented and described in detail, pulling apart those related to the detection of onshore and offshore areas affected by gas flaring (Section 3.1) by the ones concerning the emissive power sources estimation (Section 3.2) and the gas flared volumes computation (Section 3.3). All the results, aggregated at national and state scale, refer to each year of the investigated temporal period (2000–2016).

### 3.1. Detection of Flaring Sites

**Figure 5:**
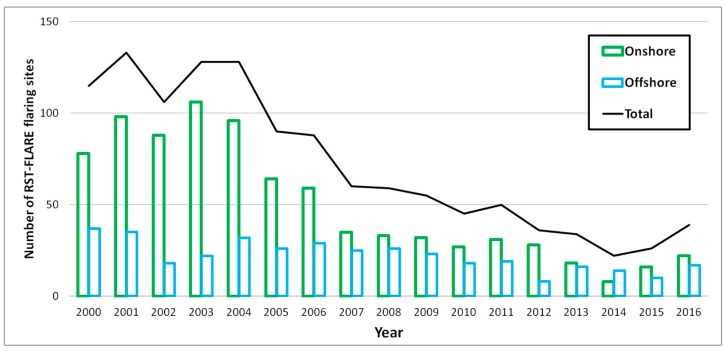
Temporal trend of total flaring sites detected applying RST-FLARE (black line) in the Niger Delta region, for each year from 2000 to 2016, as sum of onshore (green bars) and offshore (light blue bars) sites.

By applying the methodological step described in Section 2.3.1 the flaring sites active in each of the years between the 2000–2016 period in the Niger Delta basin were first identified (black line in Figure 5), discriminating also between onshore and offshore sites (green and light blue bars, respectively).

Besides the total number, also the spatial distribution of the onshore and offshore detected flaring locations among the seven states of the region has been assessed (Table 1).

Looking at these values, Rivers is the state with the greatest number of flaring sites both in the terrestrial and marine environment, followed by Delta and Bayelsa.

**Figure 6:**
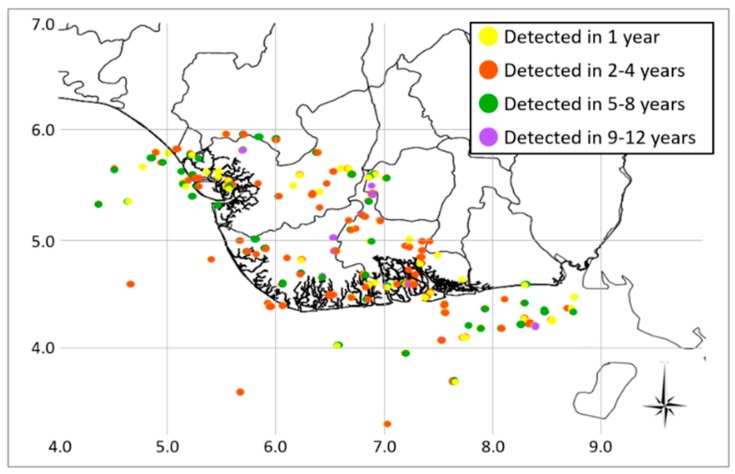
Spatial distribution of flaring sites detected by RST-FLARE in the years 2000–2016, differently colored according to their occurrence frequency.

The spatial distribution of the detected flaring sites, together with their frequency of occurrence, is reported in a map, shown in Figure 6.

The map, showing a total of 314 flaring locations (derived from the multitemporal aggregation), of which 68% (215) onshore and 32% (99) offshore, provides also information about the activity duration in order to have a general overview of the areas mostly perturbed by gas flaring. In particular, sites have been depicted with different colors on the basis of how long (in terms of years) they were detected as active within the whole investigated temporal period. By observing Figure 6, the most of sites were active in between two and four years (41%, orange dots), followed by the ones active in one year only (25%, yellow dots) and operating in five or at most eight years (25%, green dots). Only a 9% of sites has been identified as active for over 9 years (violet dots).

### 3.2. Computation of Source Emissive Power

**Figure 7:**
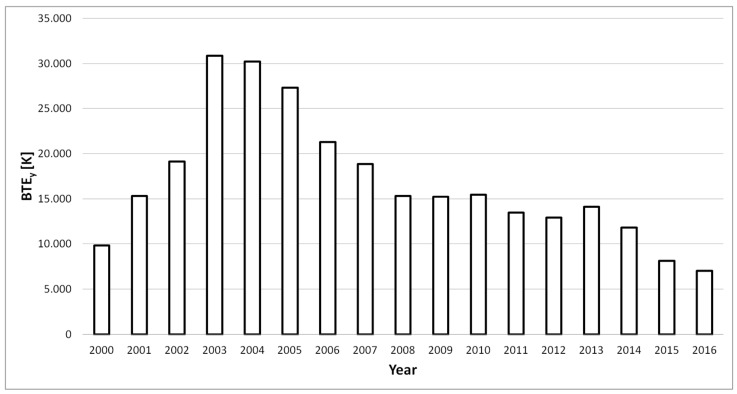
Flaring sites BTE_y_ temporal trend.

The emissive power of the flaring sites was assessed by computing the BTE (Equation (2) in Section 2.3.2). Figure 7 shows the BTE values for each year from 2000 to 2016 referred to the previously identified flaring sites (i.e., 314 pixels).

Looking at Figure 7, it is evident a great increasing of BTE from 2000 to 2003 (when the peak value was measured), followed by a continuous decreasing trend between 2004 and 2016, except for a slight increase in 2013.

**Figure 8:**
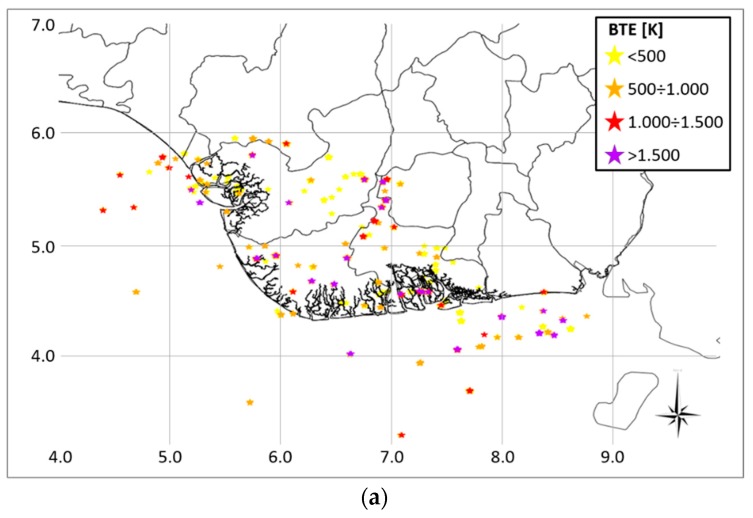

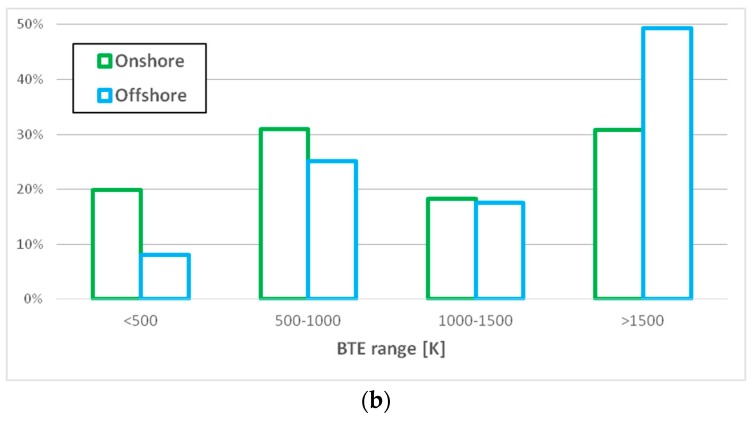
(**a**) Spatial distribution of BTE classes in the investigated states, (**b**) distribution of BTE classes between onshore and offshore.

Finally, an overview of how much flaring sites have impacted the region in the long period, in terms of BTE, is shown in Figure 8a. In such an image the BTE was classified in four classes, ranging from <500 K (the lowest level) up to >1500 K (the highest level).

For onshore areas, the BTE distribution among the four classes is quite similar, with higher intensity for 500–1000 K (31%) and >1500 K (31%) ranges (Figure 8b). On the other hand, for the offshore sites the most of BTE is higher than 1500 K (49%), with only 8% at the lowest BTE level (<500 K). The remaining belong quite similarly to the intermediate levels (25% for the second range and 17% for the third).

### 3.3. Estimation of Gas Flared Volumes

Preliminarily to the model development, we evaluated the agreement between the computed radiometric variable (i.e., BTE) and the quantity to assess (i.e., gas flared volumes). To this aim, the temporal trends of official OPEC and EIA data on gas flared volumes (Figure 2) and the BTEs (Figure 7) were compared, revealing a very good accordance, showed by a linear correlation coefficient (R) of about 82% (computed in years 2000–2014) and 84% (computed in years 2005–2014) when OPEC and EIA datasets are considered.

Basing on this satisfactory result, we coupled OPEC and EIA datasets (Figure 2) with the computed BTEs (Figure 7), developing, for each one of them, the corresponding linear regression models. For both ancillary datasets, the data up to 2013 were exploited to calibrate the models (14 data points for OPEC and 9 for EIA). The information referred to each model are shown in Table 2.

**Figure 9:**
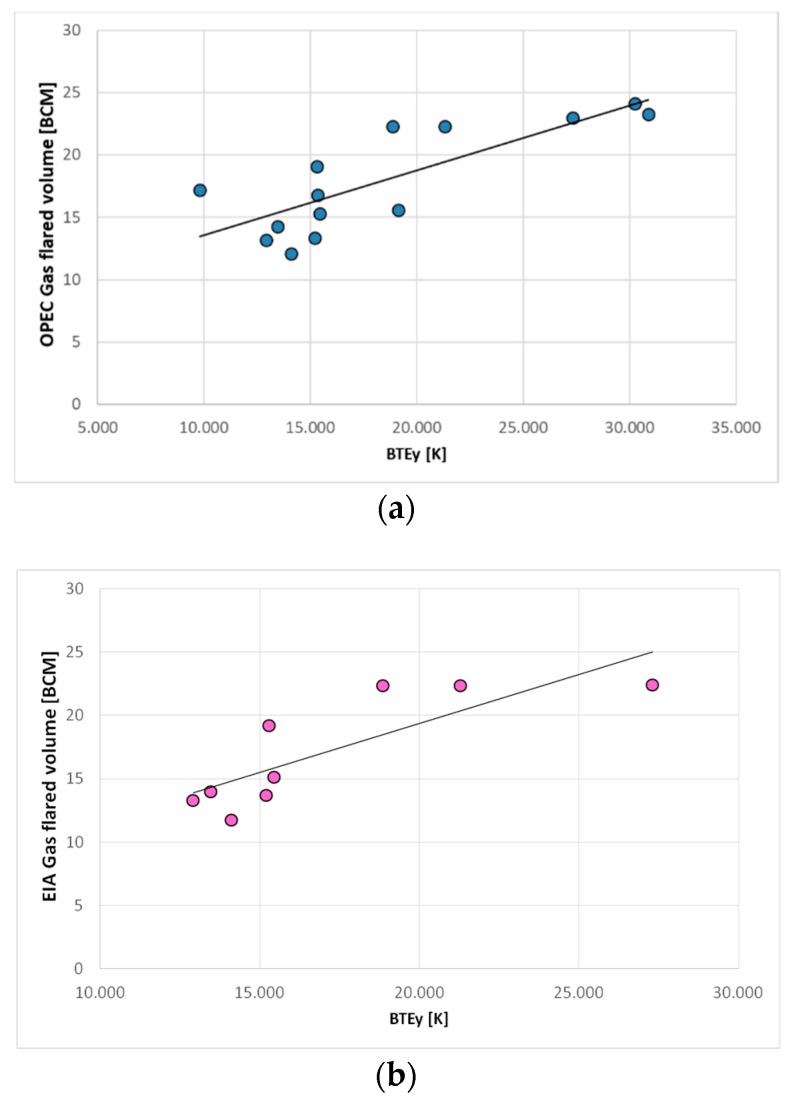
Plots of the linear regression models generated by exploiting (**a**) OPEC and (**b**) EIA dataset.

Table 2 includes, for each model, the corresponding coefficient of determination (R^2^) and the *p*-value as well as the amount of gas flared in Niger Delta estimated by the models in 2014–2016. Figure 9 show the scatter plots of both models.

## 4. Validation Analyses

Validation analyses were carried out both for flaring sites discrimination and flared volumes computation.

### 4.1. Flaring Sites

The temporal and spatial distribution of flaring sites, discussed in Section 3.1, were validated using two independent datasets (see Section 2.2).

About the first analysis, the flaring sites annually detected by RST-FLARE, in terms of temporal trend (solid line in Figure 5) and number, for onshore and offshore as well as for each state (Table 1), were compared with results achieved by [12].

**Figure 10:**
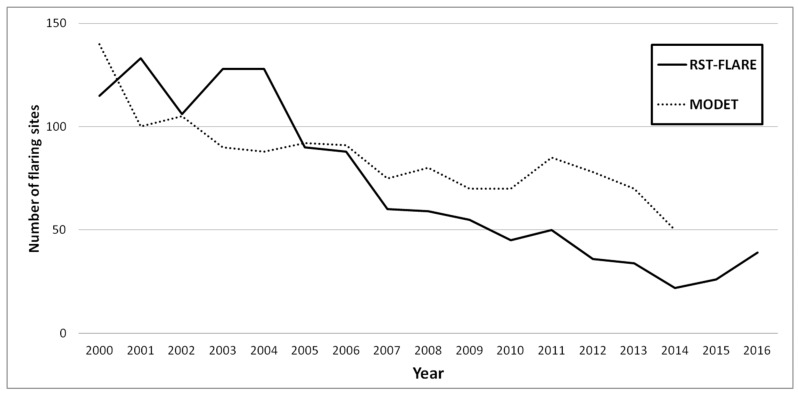
Annual number of flaring sites detected in the Niger Delta region by RST-FLARE (solid black line) and MODET (dashed black line, adapted from [12]).

By qualitatively comparing the temporal trend of flaring sites detected by the MODET algorithm (dashed black line in Figure 10, qualitatively extracted from Figure 7 in [12]) in the Niger Delta from 2000 to 2014 with the ones obtained by RST-FLARE (solid black line in Figure 10), a correlation of 74% was found.

Observing Figure 10, it is interesting to note that both algorithms show a general decreasing trend in the number of flaring sites during the whole investigated period, with a rate slightly higher for RST-FLARE than MODET. Although some discrepancies between the two series are evident, the two products are quite similar, not only in the temporal trend but also in terms of the order of magnitude of the annual detected flares.

Besides, a quantitative comparison between the spatial distributions of flaring sites detected onshore and offshore by RST-FLARE (Table 1) and MODET (Table 4 in [12]) was carried out. These algorithms identified a very similar distribution of flaring sites between onshore (68% for RST-FLARE, 70% for MODET) and offshore (32% for RST-FLARE, 30% for MODET) zones. In addition, when the flaring sites distribution across the seven Niger Delta States is compared (see values in Table 3), the agreement of the two algorithms growed up to 99% for onshore detections and 88% for the offshore ones (Table 3).

**Figure 11:**
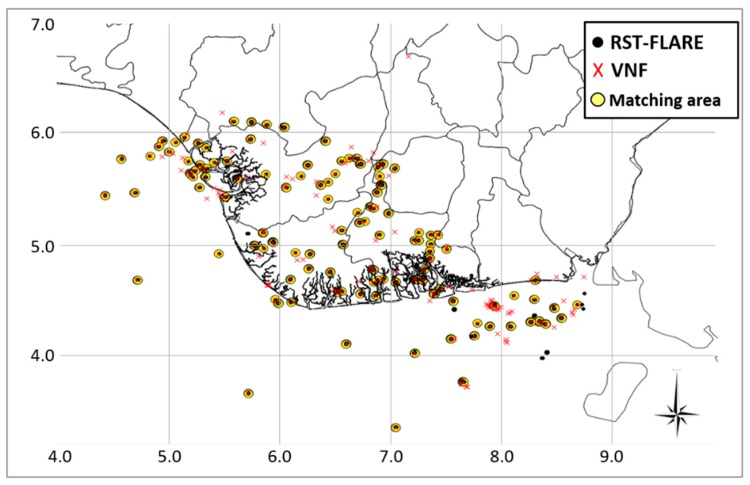
Spatial distribution of flaring sites detected by RST-FLARE (black dots), in the period 2000–2016 and VNF (red crosses), for the years 2012–2016. The yellow circles highlight the match between detected areas.

To better validate the spatial distribution of the detected flaring sites, a fully independent satellite-based map, the VNF product [7], was also used. It is worth underlining that RST-FLARE and VNF, although based on a different multi-temporal aggregation scheme for the gas flares detection (17 years long for RST-FLARE and 5 for VNF, aggregated on an annual and monthly scale, respectively), show significant differences, using different satellite data offering different spatial, spectral and temporal resolutions. To take into account such aspects, the inter-comparison was carried out defining a 1 km - buffer zone around each detection of both VNF and RST-FLARE. In Figure 11 the spatial distribution of flaring sites detected by RST-FLARE (314 pixels, black dots in the map) and VNF (205, red crosses, extracted from the SkyTruth map) is shown. In this figure, the yellow circles highlight those flaring sites identified by both algorithms within the 1 km buffer zone.

About 95% of the indicated flares have been commonly detected by the two algorithms, confirming their good agreement, in spite of the above mentioned significant differences between them.

### 4.2. Gas Flared Volumes

**Figure 12:**
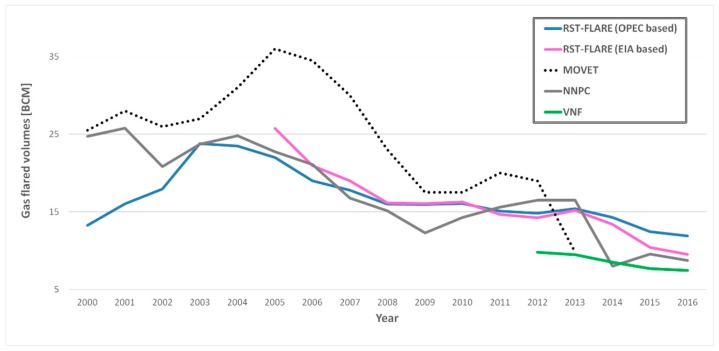
Annual gas flared volumes computed for the Niger Delta region by RST-FLARE, based on OPEC (blue line) and EIA (pink line) sources. MOVET, adapted from [12] and VNF estimates (black dotted line and green line, respectively) are also shown. The solid grey line refers to the data independently provided by NNPC.

The volumes of gas annually flared in Niger Delta were computed by the two RST-FLARE models developed using OPEC and EIA data (Table 2). These estimates, shown in Figure 12 (blue line for the OPEC-based model and pink line for the EIA one), were first compared with volumes provided by MOVET in the period 2000–2014 (black dotted line in Figure 12) and VNF in years 2012–2016 (green line in Figure 12). The NNPC data (solid grey line in Figure 12) are then used to validate the RST-FLARE products, offering the longest temporal series.

Concerning the comparison with the MOVET outputs (developed by [12] and extracted from Figure 10 in their paper), despite some discrepancies in absolute terms, the estimates derived from the two satellite algorithms show a quite good agreement: R ~ 62%, in the period 2000–2013, between MOVET and RST-FLARE based on OPEC source; R ~ 86%, in the period 2005–2013, when EIA-based RST-FLARE curve is taken into account. A high correlation (~95%) was found when RST-FLARE estimates were compared with the VNF ones, in the period 2012–2016. In this case, it is very interesting to note that both satellite algorithms highlighted a similar rate in reduction of the emitted volumes of gas flared, but with RST-FLARE absolute values almost double than the VNF ones.

When the above mentioned satellite products were compared, each one in its corresponding temporal range, with the trend of values published by the NNPC (solid gray line in Figure 12), a reasonably close correspondence was found: ~65% and ~88% for OPEC and EIA-based RST-FLARE products (in 2000–2016 and 2005–2016, respectively), 66% for MOVET (2000–2013) and 88% for VNF (2012–2016). In Table 4 the biases between NNPC values and the analyzed satellite products are reported.

Analyzing the errors in Table 4, RST-FLARE seems to offer good performances when compared to both MOVET and VNF. A mean bias in between ~16% and ~20% is committed in the annual volumes computation, when EIA and OPEC based models are employed, against the ~34% and ~25% of MOVET and VNF. RST-FLARE models are also the ones offering the best results in terms of minimum bias which, for both configurations, remains <1%. On the other hand, as far as the maximum error is considered, VNF seems the best method, assuring an error never higher than 43%.

Besides, as far as the whole time period is considered, according to the NNPC data, a total of 270.7 BMC of gas was flared between 2000–2013 in the Niger Delta states. In the same period, the volumes computed by RST-FLARE, based on OPEC data, are equal to 246.7 BCM, 9% less than NNPC, while MOVET assesses 344.8 BCM, overestimating of about 27%. RST-FLARE showed even higher performances when the EIA-based model is used: in the period 2005–2013, 158.2 BCM of burned gas were estimated, only 2% higher than the NNPC value (i.e., 154.9 BCM), compared to the 197.5 BCM estimated by MOVET, resulting in an overestimation of about 34%. Similarly, with reference to 2012–2016 period, the RST-FLARE estimates of gas flared volumes (62.7 and 68.8 BCM) were about 6% and 16% (respectively for EIA and OPEC models) higher than the NNPC value (i.e., 59.3 BCM). For this period, the VNF overestimation respect to NNPC is ~28%.

In summary, the validation analyses, carried out exploiting different kinds of independent data, both satellite and ancillary, seem to reveal the good potential of RST-FLARE in providing quite accurate information on gas flaring, especially in the long period, when compared with NNPC dataset. In all cases, the RST-FLARE EIA-based model showed the best results.

## 5. Discussion

In this paper, the RST-FLARE algorithm, aimed at detecting flaring sites and computing the volumes of gas annually flared by these sources at national scale, by processing multi-year time series of nighttime MODIS infrared imagery, is presented. In detail, its performances when applied to the Niger Delta region, an area where gas flaring associated with petroleum exploration is contributing immensely to air pollution, are shown and discussed. In general, the results achieved confirm the RST-FLARE potential in providing quite reliable information on gas flaring activity in Niger Delta. The proposed method has been validated, in terms of temporal trend and spatial distribution of flaring sites, as well as of annually emitted gas volumes at national scale, by using different independent sources. An agreement between 74% and 95% versus MODET and VNF products was found when flaring sites were detected. Using the annual BTE values in the linear regression models provided satisfactory estimates of gas flared volumes, with a mean bias in between 16% and 20%, in the annual computation, decreasing up to 2% in the long period one. With reference to the BTE metric, it is very interesting to note its good agreement with the information reported in Section 3.1, referred to the history of Nigerian oil and natural gas productions as well as with the variation of flaring sites (black line in Figure 5). Regarding this aspect, it is very interesting to note that on years 2014–2016, a reversal behavior of gas flare numbers and BTEs is observed. In these years, in fact, the emissive power (BTE) decreases of about 68% while the number of flaring sites increases of about 77%. This seems to confirm the headway into drastically reducing the flaring of associated gas in Niger Delta, achieved through aggressive gas commercialization as said the NNPC Group General Manager [42]. Also online information, referring to the evolution of Nigeria gas flaring [11,43,44] are in line with BTE estimates confirming the decreasing of such a phenomenon in the Niger Delta basin. As reported by GGFR, in Nigeria alone gas flaring amounts to about 23 BCM per annum in over 100 flare sites, constituting over 13% of global gas flaring [44]. In the recent years, Nigeria, traditionally one of the highest flaring countries, has made significant progress to enforce zero gas flaring policy, reducing it by 18% since 2013, to less than 8 BCM in 2015 [44].

Although these satisfactory achievements, residual errors and inaccuracies have been identified and are briefly discussed in the following.

**Figure 13:**
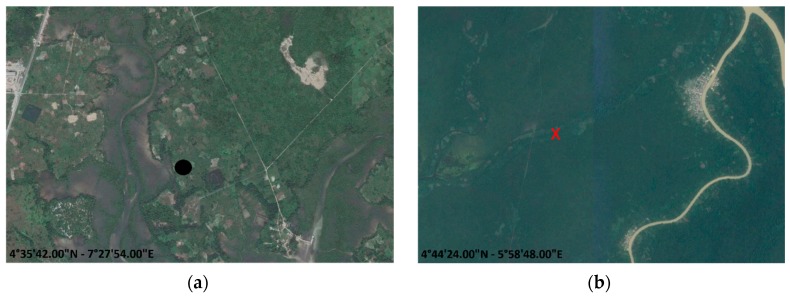
Two examples of false positives by: (**a**) RST-FLARE; (**b**) VNF. In background are GoogleEarth^®^ images of (**a**) 19/12/2013 and (**b**) 23/12/2015. The visual inspection has been carried out for the whole temporal period exploiting the GoogleEarth timeline tool (clock button).

(a) The first cause of inaccuracy in effectively discriminating flaring sites in the Niger Delta region could come from the use of the MIR signals. Considering that gas flares typically operate at temperatures >1400 K, with a peak spectral radiant output occurring in the SWIR region [7], at MIR wavelengths their emissions are significantly minor (about one third of the signals emitted in the SWIR region). This could result in an under estimation of flare spectral response, especially for the hottest sources [7,25,26]. However, RST-FLARE, thanks to its differential and self-adaptive nature (i.e., exploiting locally derived, in space and time domains, rather than fixed thresholds to identify signals above background), enables the identification of flaring sites over the considered long-period with a good accuracy level when compared with both ancillary data and satellite methods.
  Besides the comparison to independent data sets, even the analysis of the temporal trend of the detected flaring sites (black line in Figure 5), seem to confirm the accuracy of the results, allowing for a reconstruction of the flaring history in the region. The Niger Delta basin is strongly affected by onshore gas flaring, with a number of flaring sites almost double respect to the ones identified offshore. In both cases, an overall general reduction in the number of active flare sites between 2000 and 2014 was observed, with a significant decreasing trend that started in 2003 onshore and in 2004 offshore, respectively. Especially for the onshore sites, the general downward trend in the number of flares is due to several causes, mainly related to the operations carried out since 2002 for the installation of associated gas gathering infrastructures at various oilfields [12,45,46,47]. Moreover, the Niger Delta militants’ actions occurred between 2006 and 2009, which severely disrupted oil and gas production in the region, may explain the observed reduction in total number of flaring sites in the same period [48]. Since 2014 (for onshore) and 2015 (for offshore), a new increasing in flaring sites seems to be started (of about 64% for land and 36% for sea areas). No information on gas flaring sites, both from official sources and independent satellite methods, have been found to explain such a significant relative variation. It can be probably due to new investments in oil plants equipped with modern infrastructures able to collect, treat, transport and utilize the associated gases. Of course, further analyses have to be carried out to assess these results. Besides, the satisfactory agreement with MODET (between in 74% and 95%) strengthen the RST achievements, confirming the high variability among the years in the number of operational flares, even if detected with a different level of accuracy by the two algorithms. The different aggregation scheme, bi-monthly (only December and January) over 15 years (2000–2014) for MODET, monthly (for each calendar month) for the 2000–2016 period for RST-FLARE, may be one of the possible causes of the observed discrepancies together with the different detection methods and thresholds.
  Concerning the flaring sites localization, RST-FLARE shows again a very good agreement (95% of spatial matching) when compared to results derived by means of VNF, a fully independent product based on VIIRS data offering improved spectral (with a SWIR band capability) and spatial (~750 m of pixel size in SWIR) resolution. Such a cross-validation between two independent satellite-based products reveals to be helpful in a twofold perspective: (i) common detections strengthened the accuracy of each system in providing reliable and consistent information; (ii) on the other hand, unmatched detections might reveal possible false positives (a couple of examples are shown in Figure 13), allowing for further “fine tunings” and optimization of the products.
(b) A further possible issue in the flaring sites characterization may be due to the variability in sampling and cloud cover, which could affect the RST-FLARE results consistence between years. In order to reduce these effects, a coefficient *α_j_*_,*y*_ has been defined and computed for each of the detected flaring sites within the single year. It allows normalizing, between investigated years, the source emissive power (quantified in terms of Brightness Temperature Excess, BTE, see Equation (2)) according to the imagery sampling caused by the cloud coverage and the satellite view geometry.
(c) Additionally, the MIR MODIS signal, used in the BTE metric computation, may be affected by the atmosphere. Although a number of papers in literature [49,50] do not use atmospherically corrected MODIS MIR radiances and, more recently, [51] suggested considering the atmospheric transmittance of the 4-μm channel (MODIS band 22) equal to 1 when computing the Collection 6 MODIS fire product, atmospheric effects might be not completely negligible. However, the RST-FLARE configuration partially takes into account and mitigates the atmospheric effects impact by using the <BT20*_j_*_,*bg*_> spatial mean as reference value for BTEs computation as well as the ALICE index. For its construction, *μ*_20_(*x*,*y*) is representative of average conditions in terms of the above-cited noise sources, therefore, if in one image their contribution is quite relevant, producing a significant attenuation in BT20, the corresponding BTE will result lower. The ALICE, using the temporal mean, enables to further reduce the impact of signal variability in the image at hand due to possible noise sources, such as the geometry of observation and atmospheric contributions. Ref. [12] provided a similar indication, defining their bi-monthly composite aggregation scheme a practical and effective atmospheric correction method.

## 6. Conclusions

This study presents the RST-FLARE performances in characterizing the Niger Delta region gas flaring activity, using multi-year time series of nighttime infrared (both MIR and TIR) MODIS data.

The main achievements are summarized as follows:

(i) sites affected by flaring activity are automatically identified, at different temporal scales (both annual and multitemporal), using a standardized variable index measuring the local variation of infrared radiation from flare;
(ii) the volumes of gas annually emitted, at national scale, are computed by developing a regression model using the Brightness Temperature Excess (BTE), measuring the difference between the 4-μm source emission and its surrounding expected background, as well as official data on gas volumes emitted by the plants to quantify the emissive power of the hot sources;
(iii) the long term variation in flaring activity, in terms of flaring sites and flared volumes, is evaluated considering annual estimates within the 17-year investigated period.

In summary, based on these achievements, RST-FLARE seems to be a reliable semi-automatic tool to be used to identify gas flaring sites, to estimate gas flared volumes and to analyze possible spatiotemporal dynamics at flaring sites. With this capability, and thanks to its exportability on different satellite data and geographic area, the proposed approach can be implemented to whatever country to investigate gas flaring activity provided that a long-term time series of useful (in terms of spectral, spatial and temporal resolution) satellite data and independent official data on gas flared volumes are available. Therefore, results specific of each considered region of interest will be produced.

Works in progress are currently focused on the calibration of a new version of RST-FLARE aimed at exploiting the great potential of SWIR VIIRS (since 2012) band, which, thanks to its spectral and spatial resolutions, should improve RST performances in detecting flares and measuring their radiant power. In the next future, also the Sea and Land Surface Temperature Radiometer (SLSTR) SWIR bands capability will be explored as soon as a consistent dataset will be available for RST implementation.

## Figures and Tables

**Table 1:** Distribution of flaring sites in the Niger Delta states between onshore and offshore.

State	N. Detected Flaring Sites		
	Onshore	Offshore	Total
1. Edo	19	0	19
2. Delta	56	23	79
3. Bayelsa	54	15	69
4. Imo	12	0	12
5. Rivers	70	34	104
6. Abia	1	0	1
7. Akwa Ibom	3	27	30
**Total**	**215**	**99**	**314**

**Table 2:** Regression models data.

Source	Temporal Range	Regression Model Equation	R^2^	*p*-Value	Year of Estimation	Gas Flared Volume [BCM]
OPEC	2000–2013	Gas flared [BCM] = 0.0005*BTE + 9.401	65%	<0.01	2014	14.3
					2015	12.4
					2016	11.9
EIA	2005–2013	Gas flared [BCM] = 0.0008*BTE + 3.902	67%	<0.01	2014	13.4
					2015	10.4
					2016	9.5

**Table 3:** Distribution of flaring sites detected, onshore and offshore, by RST-FLARE and MODET (source: Table 4 in [12]) across the seven Niger Delta states.

State	Onshore		Offshore	
	RST-FLARE	MODET	RST-FLARE	MODET
1. Edo	19	19	0	0
2. Delta	56	51	23	19
3. Bayelsa	54	41	15	12
4. Imo	12	11	0	0
5. Rivers	70	56	34	16
6. Abia	1	1	0	0
7. Akwa Ibom	3	6	27	30
**Total**	**215**	**185**	**99**	**77**

**Table 4:** Biases between NNPC and RST-FLARE, MOVET and VNF estimates.

	Max Bias	Min Bias	Mean Bias
**RST-FLARE OPEC-based**	78.4%	0.3%	19.7%
**RST-FLARE EIA-based**	67.0%	0.8%	15.9%
**MOVET**	78.9%	3.2%	34.1%
**VNF**	42.5%	6.2%	24.9%
